# Hospital and Institutional News

**Published:** 1916-11-18

**Authors:** 


### HOSPITAL AND INSTITUTIONAL NEWS.

### A BRITISH RED CROSS UNIT FOR RUMANIA.

A Red Cross unit organised by the Joint War Committee of the British Red Cross Society and the Order of St. John of Jerusalem has started for Rumania, presumably via Russia. The unit has been provided as a gift from the miners of Great Britain, who voted funds for its formation and equipment. The unit is provided with four motor ambulances and a touring car, and is commanded by Dr. O'Leary. There are three other surgeons—all Fellows of the English or Edinburgh Colleges of Surgeons—and a dental surgeon; a matron and four nurses; two (woman) cooks; five (male) orderlies; a radiographer; a storekeeper; a carpenter; a transport officer and five drivers. The Joint War Committee has by this time such an accumulated fund of experience on war hospitals that it may be assumed there is some special need in Rumania for a hospital rich in experienced surgeons. To the uninitiated it might appear that four nurses and five orderlies are not enough staff to allot to four doctors, three of whom are surgeons; or else that the doctors are going to have an uncommonly easy time, which means a waste of surgical skill when our own Army can do with all the surgeons available. Very likely this nursing staff is to be supplemented pretty largely by local supplies when the hospital settles down in Rumania; and in any case the unit will be a visible and tangible proof to the Rumanians of the sympathy and support which Great Britain extends to them in their hour of trial.

### MORE WOMEN MEDICAL OFFICERS FOR THE ARMY.

The War Office are appealing for fifty more medical women for service at home in military hospitals, with the status of lieutenant in the Army, pay at the rate of one pound a day and allowances, and promotion in rank in the usual way (to captain at the end of one year, presumably). It is stated that more than one hundred women are already serving their country in this way. There are between seventy and eighty at Malta, including surgeons, physicians, pathologists, radiographers, anæsthetists, dental surgeons, and other specialists. According to an article in the *Daily Graphic*, dealing with the work of the medical women at the Endell Street Military Hospital, there are fifteen doctors employed there to attend on six hundred and seventy patients. There are four who rank as majors (the highest rank yet granted to a medical woman), including Mrs. Garrett Anderson and Miss Flora Murray. At first sight fifteen doctors might seem a too liberal allowance for the number of beds. But it is said that only eight are employed on the daily routine work; doubtless the others are specialists of various kinds who devote only part of the day to this work. Even then, allowing for the kind of cases to be encountered in military hospitals in England, the hospital can hardly be reckoned understaffed. It is, at least, a gratifying tribute to medical women's work that the War Office want more of them.

### SIR SAM HUGHES'S ALLEGATIONS ABOUT THE TREATMENT OF WOUNDED CANADIANS.

Some very wild allegations have been made at Toronto by Sir Sam Hughes, lately the Canadian Minister of Militia. So far as they concern the Army Medical Department and the treatment of wounded Canadians, the fullest proof of these assertions will be required before they are likely to receive any credence in this country: with the similar charges in respect of equipment and management of the Canadian fighting forces by the British authorities, the hospital world as such is not concerned. According to Reuter, Sir Sam Hughes has said that thousands of Canadians have lost months and sometimes a year of time in hospital when they should have been back in the trenches; and the allegations are that this has been due to the fact that the patients have been in British, instead of segregated in Canadian, hospitals, and that first-year medical men have been allowed to operate on Canadians instead of surgeons from the Dominion. Frankly, we do not believe that this tirade has any serious foundation in fact. The responsibility incurred by a man in Sir Sam Hughes's position who makes charges such as these is so serious that we hesitate to suggest that he is insincere: we prefer to hope and believe that he is misinformed. If this is so, all we can say is that he has not deserved well of the State by this utterance at Toronto. The latest information is that Sir Sam Hughes has resigned his position in the Ministry, at the request of Sir Robert Borden, the Prime Minister. This news confirms us in the views above expressed, for it seems to show that Canada either attaches no credence to Sir S. Hughes's wild talk, or regards him as actuated by unpatriotic motives.

### TWO VACANT HOSPITAL SECRETARYSHIPS.

It is not often that two vacancies for secretaries of hospitals in London occur at the same time, although not infrequently, by reason of promotion, one follows another. The Middlesex Hospital is advertising for a Secretary-Superintendent to fill the place of the late Mr. Clare Melhado; the salary offered is £750 per annum, and candidates, who must be members of the Church of England and between the ages of thirty and forty-five, must send in their applications not later than the 24th inst. For a commencement the emolument specified appears to be reasonable, and is sufficiently tempting to attract some strong candidates if the potential secretary is not to be found within the four walls of the Middlesex. As much cannot be said for the salary offered at the London Temperance Hospital, an institution of 116 beds, with an expenditure of over £10,000. We are sure it is short-sighted policy to attempt to get the right man for £250 a year, and if it is not too late we urge the committee to reconsider this point. That body has had long enough to reflect upon what is required, for Mr. A. W. Bodger has been dead for many months, and the vacancy caused by that regretted event has apparently not hitherto been filled. It is announced that candidates must be ineligible for military service, preferably experienced in hospital or institutional work, and total abstainers. The closing date for applications is not announced. At both institutions we should have liked to see a preference stated for disabled soldiers, other things being equal. Perhaps that goes without saying: we hope so, at any rate.

### THE DIPHTHERIA SCARE AGAIN.

A few weeks ago certain organs of the daily press caused a certain degree of panic amongst nervous people by asserting, contrary to the facts, that a serious epidemic of diphtheria was raging in London. The figures, taken from the returns of the Metropolitan Asylums Board Hospitals, of admissions for this disease were quoted in support of the assertion; and to those who do not realise what a vast population London contains, and did not know what the normal figures of sporadic diphtheria cases ordinarily are, these statistics may well have sounded disturbing. As a matter of fact the incidence of diphtheria at that time was very much what it always is in early autumn; and the panic was artificial and unnecessary. It soon passed over; but we note with regret that even still there are attempts going on to revive it, though we can scarcely understand why any responsible newspaper editor lends his columns for such an unworthy purpose. One such scaremongering article, which appeared on November 10, reads almost as if it was a forgotten bit of "copy," some weeks old, being used to fill a vacant column; for it actually suggests that the lessened watering of the streets in the "recent summer" may be responsible for the (alleged) outbreak. The author of this pronouncement can hardly have been out of doors in London since the end of September if he really thinks (as he says) that the drains have not been sufficiently flushed and that this has contributed to the spread of diphtheria. But, apart from the gravity of spreading groundless alarm at any time, surely editorial responsibility in times like these makes such an offence doubly culpable.

### WELSH NATIONAL MEMORIAL REPLIES TO CRITICISM.

Overt and covert grumblings have been made against the Welsh National Memorial Association almost ever since its anti-phthisis crusade was organised. Some counties and medical men seem to be jealous of it, there have been complaints of bad organisation and so forth, but it has not been easy to trace them to their source, or to gauge the amount of substance behind them. The general director, Mr. D. S. Evans, has therefore done a useful piece of work in summarising the Association's achievements since October 1, 1914. We append his principal statements, and if sins of omission are to be alleged our columns are open to their responsible statement, for nothing can be worse for any public body than an attitude of discontent or suspicion which does not crystallise itself. Mr. Evans says that in the past two years 6,000 patients have received *institutional* treatment; the adjective here is a very important one. Since 1912 the number of beds for consumptives has risen from 31 to over 1,000. Since the beginning of the war some 350 soldiers have passed through the Association's hands. These figures, brief as they are, do not meet the questions: What good is done? What is the percentage of "cures" and "improved cases"? On this head Dr. Patterson, the well-known chief medical officer of the Association, has defended a wise reticence. He said that final results should not be considered during a shorter period than five years. This period is not passed yet, but the material is being gathered every day. We only hope that "following-up" has been common and careful enough to make the coming statistics complete.

### THE REDDITCH SMALLWOOD HOSPITAL.

The Hospital Saturday Committee of this hospital held a meeting recently at which the honorary secretary, Mr. W. Hill, reported on the result of his endeavours to obtain increased support for the hospital from the representatives of sick clubs and benefit committees at the large works in the neighbourhood. Some of these representatives of the workers promised increased subscriptions, some are considering the matter, and some have definitely refused: the increased sum promised amounts to but £50 a year, barely a third of the minimum increase on which the hospital can be kept going on its present footing. There was a long discussion on the report, the general tone of which was disappointment at the small result of the appeal. One of the points taken was that there has been a large increase of female labour in the neighbouring works, but that the women workers (though they use the hospital accommodation even more, proportionately, than men do) have as yet no corporate sense of their duty and advantage in maintaining it at a high level of efficiency. In the end a special sub-committee was appointed to go further into the matter; possibly the upshot of the discussion, or of its publication in the local press, may be to stimulate the workers to a due sense of their responsibilities towards the Redditch Smallwood Hospital and its Saturday Fund.

### INFANT CONSULTATIONS AT QUEEN CHARLOTTE'S LYING-IN HOSPITAL.

It was decided some time ago to start an Infant Consultation Centre at Queen Charlotte's Hospital; and the managers are now advertising for a physician to take charge of the department, which is evidently to be opened shortly. By a curious coincidence the conditions as to qualification for this post show a breaking away from London tradition very much along the lines quite independently suggested in our leading article this week. Candidates, who may be male or female, must be graduates in medicine of a University of the United Kingdom; or Fellows or Members of the Royal College of Physicians of London; or Fellows of the Royal College of Surgeons of England. We do not see any provision in the conditions, as advertised, restricting the appointment to those over military age (in the case of men), nor one making it of a temporary nature until after the war. This seems to be a mistake: hospital staff appointments should not be made on a permanent basis at present, for otherwise injustice may be and will be done to those who are serving in the Royal Army Medical Corps and have no opportunity of sending in their applications.

### A QUESTION OF PRINCIPLE AT DUNFERMLINE.

Some remarkable evidence has been tendered (according to the report in the *Aberdeen Journal*) at an inquest held at Dunfermline Sheriff Court on November 9. Briefly, one John Inglis, a joiner, died under chloroform at the Dunfermline and West Fife Hospital while being operated on for an injury to one of his fingers. The administrator was a junior nurse, who gave the anæsthetic to the patient in the presence of the surgeon performing the operation. It is generally held in this country that in such a case the operator is responsible for the administration, and that the practice is one to be avoided whenever possible. In the United States it is not an uncommon practice for a nurse to administer ether; but even there, we believe, they would be somewhat shy of entrusting such an unqualified administrator with chloroform. The surgeon told the Court that at many hospitals in Scotland nurses administer chloroform, that he personally approves of the plan, and that "doctors are rather inclined to be careless with a very dangerous drug." It is to be hoped that he is misinformed as to the extent to which nurses (and junior nurses at that) give chloroform in Scottish hospitals, and that his low opinion of Scottish anæsthetists is not shared by many other surgeons across the Border. Admittedly during the present stress it may sometimes, or even often, be necessary to entrust anæsthetics to nurses, especially in small country towns. But in such cases experienced matrons or sisters should alone be employed, and the anæsthetic used should be ether, not chloroform. To his view that nurses are better suited to the handling of dangerous anæsthetic drugs than are qualified practitioners it is impossible for us to subscribe.

### THE DUNDEE ASYLUM IMPASSE.

A fortnight ago allusion was made in these columns to the extraordinarily severe censure passed by the Scottish Board of Control on the management of the Gowrie House Asylum, Dundee (The Hospital, November 4, 1916; p. 80). As was then mentioned, the Board of Control selected the medical superintendent for their especial censure, and practically invited the Local Board of Control (the Parish Council) to dismiss him. This course the Local Board has not followed: by a vote of fifteen to ten they have decided against this drastic course, in favour of the milder measure of censuring him. The Dundee local press has been ringing with the whole scandal for some time, and a lot of abuse has been hurled at the heads of the Parish Council, both before and after the decision in the matter was taken. Looking at the dispute from a distance there seems little need to import into the affair (as some of the local journalists have done) insinuations of "graft." But the Local Board of Control do appear to have overlooked or neglected one very important point in their policy of amnesty towards their medical officer. It is this: that at the inquiry held by the Scottish Board of Control most of the evidence which satisfied the latter that grave irregularities had been committed was given by nurses and other subordinates of the medical officer. The position of these witnesses becomes so awkward, if their superior is kept in office, that virtually they will all have to resign. It seems very hard that an act of mercy to an erring individual should entail, as it necessarily must in this particular case, a gross hardship on several other just persons which need no repentance; and the whole situation is so topsy-turvy that it would be no surprise to hear of the Board of Control being moved to further interference.

### SANATORIUM BUILDINGS AT £12 PER BED.

Since the Treasury has forbidden, during the war, the spending of money on most building schemes, plans for new institutions and extensions have alike been at a standstill. For instance, the Swansea and District Sanatorium Committee has not held a full committee meeting for two years. The cost of the proposed sanatorium was originally £200 per bed. A movement is now suggested in favour of erecting a temporary building at a cost of £20 per bed. Dr. Clifford, who has gone into the question, lately declared that he knew a girls' school in Swansea which, built of wood, had been standing for twenty years. He also instanced an institution in Surrey, which he described as built on Australian lines, that had cost £12 per bed, but he added that in Swansea, owing to the price of land, such a building would probably cost about £30 per bed. The future of hospital architecture may lie in the development of temporary buildings, and all who remember Dr. Wrench's polemics at the end of his "Life of Lord Lister" will observe how much may be said in favour of such a change. But a suitable sanatorium at £12 per bed, in this country, appears too cheap to be practicable. Dr. Clifford was definite, however, and presumably had carefully checked his figures.

### MISS BECKETT DENISON'S LEGACY TO THE DONCASTER ROYAL INFIRMARY.

Mr. Edwin T. Hall, of Bedford Square, London, has been asked by the governors of the Doncaster Royal Infirmary to visit and report upon the suitability of the hall and grounds left to the institution by the late Miss Beckett Denison as the site of the new hospital. Mr. Hall, who has drawn the plans of a number of large modern hospitals, was also requested to inspect and report upon an alternative site. When his report is received it is the intention of the governors to call together their supporters throughout the district and formulate a scheme for the erection of a thoroughly up-to-date, fully equipped, and adequately endowed hospital. The legacy of £500 left by Miss Denison is being used in part payment of the estate duty on the hall and grounds, which is considerable, as its value is put by the Land Valuation Department at £9,600.

### GIFTS IN KIND TO HOSPITALS.

There is more than one way in which people may help hospitals, and though appeals for financial assistance sometimes fall short of the desired result, it is seldom that requests for help in kind prove anything but most gratifying. A scheme lately put into operation by the secretary of the Mansfield Hospital may give an idea to other people connected with hospital management. The aim was to provision the institution as far as possible for the winter months, and a "produce week" was organised, during which the farmers and gardeners of the neighbourhood were asked to deliver at the hospital, or send by rail to Mansfield station, gifts of potatoes, carrots, turnips, beetroot, tomatoes, apples, stewing pears, poultry, game, rabbits, butter, cheese, eggs, and such-like commodities. Depôts for the receipt of produce were opened in the villages throughout the area served by the hospital, and the Mansfield branch of the Nottinghamshire Farmers' Union heartily seconded the efforts of the hospital to gather together a goodly stock of useful eatables. In other places "pound days" for specific articles have shown that there are reserves of generosity which only need to be tapped. At Bedford, for example, 3,500 lb. of jam was soon forthcoming for the Red Cross hospitals in response to an appeal, and similar results have been achieved elsewhere.

### SHORT SHRIFT FOR PRO-GERMANS.

The Birmingham Public Health Committee, meeting on Friday last, refused to ratify the appointment, by a sub-committee, of Dr. Ella Scarlett-Synge as second assistant medical officer at the Corporation's fever hospital at Little Bromwich, at a salary of £300 per annum. Dr. Scarlett-Synge should have taken up her appointment on the previous Tuesday. The circumstances of the case are peculiar and have aroused much comment. When the vacancy at Little Bromwich occurred the Corporation advertised it, but only received two replies, both from women doctors. Mrs. Scarlett-Synge was selected, but when it became known that she had visited Germany, had attempted to justify the conditions prevailing in the internment camps there, and published a pamphlet which is considered to be anti-British, great resentment was expressed in loyal Birmingham, and a strong feeling against her being permitted to take up a public appointment manifested itself. When she attended before the sub-committee for an interview nothing was known about her German sympathies, and Dr. Robertson, the city medical officer of health, last week stated that he then was unaware of anything connected with the applicant, apart from her professional career. As a result of the agitation the appointment has thus been cancelled by the health committee.

### AN OFFICIAL NATIONAL INSURANCE HANDBOOK.

One of the principal difficulties in the way of obtaining exact and authoritative information on any point of detail connected with the administration of National Health Insurance has been the bewildering mass of Regulations and Orders which have been issued from time to time by the various authorities appointed under the National Insurance Acts. Of all shapes and sizes, many now obsolete, others only partially repealed, and in all too numerous for even the titles to be readily remembered, these Regulations and Orders have provided a veritable maze for all but the most highly trained permanent National Insurance officials. This state of affairs has now happily been remedied by the issue of an official Red-book under the title "The Statutes, Regulations, and Orders relating to National Health Insurance, with Notes, Cross-references, and an Index." Some idea of the voluminous character of the official publications can be gathered from the fact that the codification of those at present in full force occupies no less than 679 closely printed pages. Not the least useful portion of the new publication is the index, which in itself extends to thirty-four pages. The price printed on the title-page is six shillings, but the Insurance authorities were evidently so desirous of securing a large circulation that a rubber stamp has been used to obliterate the printed figure and to substitute the more modest charge of two shillings and sixpence.

### A GODALMING DOCTOR WHO ATTENDED WELLINGTON.

A London newspaper points out that Dr. Henry F. Holland, of Godalming, who attended the great Duke of Wellington, is still alive, hale and hearty. The Iron Duke died in 1852, sixty-four years ago, and Dr. Holland was a young Charing Cross graduate at that time. He had been called in to attend the Duke's butler in 1851, for a broken leg due to a cab accident; and the old Duke took such a fancy to the young man that he placed himself in the latter's hands not long afterwards when he suffered from an attack of broncho-pneumonia. Dr. Holland retired from practice many years ago and settled down in his native town of Godalming, where he is a member of the corporation. The correspondent who recalls these incidents remarks that no one seeing Dr. Holland's alert and sturdy figure in Godalming streets would credit him with his eighty-seven years. Long may Wellington's doctor flourish, to see England in as proud a position as that to which the grand old Duke lifted her a hundred years ago!

### THE COCAINE COMMITTEE.

The prevalent habit of evading Ministerial or Cabinet responsibility on any controversial or delicate topic by handing it over to a Committee has already attained dimensions which some economists consider detrimental to the State. Without expressing any opinion on this wider aspect of current Parliamentary procedure, it does seem as if the Home Secretary is showing a want of backbone by appointing one more of these Committees to deal with the question of the supply of cocaine for dental purposes. The subject is so comparatively trivial, so eminently one for the Minister's decision after consultation with his responsible medical advisers, that to appoint a Special Committee is much on a par with the traditional use of a sledge-hammer to kill a butterfly. It is true that stocks of some of the non-toxic substitutes which have largely and rightly superseded cocaine in dentistry have run low, because they are manufactured in enemy countries. But this is not true of all of them, and we cannot believe that any but a good purpose would be served by prohibiting cocaine in dental surgery altogether. In any case, we hope to hear in due course that unregistered dentists are no longer to be supplied with this dangerous drug. Incidentally, the Committee consists of three members of Parliament, a County Court Judge, and a Professor of Physiology, who is not a qualified medical man. We doubt whether a Committee so constituted will come to any better decision than the Home Secretary could himself have arrived at by a little study and a little courage.

### "INASMUCH."

No larger appeal than the one word which heads this paragraph, and consequently none swifter to its goal, is given in a leaflet published by Guy's Hospital on behalf of the children who attend the out-patient department, for whom a sad disappointment seems to threaten: the abandonment this year of the tea which has been given them for nineteen years in succession on Christmas Day. The difficulty is a real one. For the first time this year the students and nurses who used to act as collectors for this fund are not available. They are mostly away at the war. The guests are drawn from the boys and girls, between six and eleven years old, who have attended the out-patient department during the year, or are recommended by the district nurses from personal knowledge of their homes. The children as a rule number about 600, but unless the public takes up the work which the students and nurses have left behind them, these poorest of London's poor children will not have their customary Christmas treat. A hospital tea, as the public has realised by this time, is not an affair of bread and butter. It is a collection of cakes and jam and fruit and crackers, with enough bread and butter thrown in to keep up an air of respectability. It also includes an entertainment and presents for each guest. The cessation of such a "tea" would be a serious disappointment, not to say a calamity. So please take the place of the students and nurses and help to provide the £80 required.

### RAIDING THE POULTRY FARMER.

Whether from the high price of food or the limitations of the fowl, the poultry industry, in so far as its main concern is the production of eggs, is at best a struggling one. There are a few notable instances of egg farms which have been made to pay handsome profits to their owners, but the owners seemed to have a special gift for this class of work, and usually, in addition to being first-rate men of business have mastered the art whereby the products of their own particular farm can command fancy prices. One is almost tempted to say that such men could make any business pay, and that it is in spite rather than by means of the fowls that eggs on their farms are produced at a profit. At all events, with the run of poultry-keepers such success is not to be found. Consequently now, when eggs are scarce, hospitals greatly in need of them, and foodstuffs more expensive than ever, it is hardly fair to ask these poultry farmers for gifts of eggs to the hospitals. Cases have been reported to us where such requests are made with sufficient frequency as to become a regular tax on this struggling industry, and it is time to remind those responsible for their issue that the name of charity must not be abused. That special funds should be raised for the purchase of new-laid eggs is one thing, to demand their free gift from the producers is another. We hold no brief for this industry, but it is common knowledge that it is not one of those which can afford to contribute gifts in kind from its own stock.

### THE FALLACY OF THE OLD DEBT.

Public support seems to be vigorous where the Longton Cottage Hospital, Staffordshire, is concerned. The treasurer again has a handsome balance in hand to carry forward, and he and the secretary, Mr. William Atkins, are to be congratulated on the fact that the income increased by over £500 last year. It is true that the expenditure rose by an almost identically similar sum, but to be able to show an income increasing in sufficient proportion to meet the heavy rise in prices is a fact to be proud of in these hard times. The happy result is that the substantial balance in hand is virtually undiminished. This seems an instance in disproof of the theory that a hospital should always be in low financial water, so as to have a grievance to plead, and the attraction of beggary to offer. More support is to be found in appealing for means to extend equipment or increase the work which an institution is capable of undertaking than in any appeal to wipe out old debts, or to pay off ancient loans. A hospital should have much to ask for—means, in fact, to complete a carefully planned policy of growth. What may be termed the old-debt fallacy is always worth exposing, and we are glad to see that the supporters of Longton Cottage Hospital are only encouraged by a balance in hand to give their support generously enough for this to be kept till a use be found for it in raising the standard, or enlarging the scope, of the hospital's existing departments.

### WAR CHARITIES.

The first list of war charities published by the Charity Commissioners contains the names of 953 organisations, the nature of whose work brings them within the recent Act. These charities have been registered with the proper authorities in all parts of England and Wales; there are fifty-three registrations in London, eighteen in Manchester, and sixteen in Liverpool. Bristol appears to be particularly active in charitable work in connection with the war, having twenty-two names to its credit. Many of the charities recorded in the list appear to be, in reality, branches of larger organisations; for instance, local branches of the Red Cross Society are quite numerous, and Belgian Relief Funds appear with great frequency. Some of the names throw but little light on the nature of the work done; for instance, what may be the particular charitable activities of the Sunningdale Ladies' Golf Club or of a society named "Inky Imp"? Gradations of the extent of the work must be judged from small or no data as the charities range in importance from the National Relief Fund to the Usk Jumble Sale Committee. The first list is extensive, yet we learn that under a new regulation charities whose areas are small and are of a very temporary nature may, in the discretion of a registration authority, be exempted.

### THE LENGTH OF APPEALS.

One of the most successful and curious commercial discoveries of recent times was that made by a patent-medicine millionaire, who used to write his own advertisements—the discovery that an advertisement could hardly be too long. He made it plain that the successful quack is he who wraps the consultation round the bottle in several closely printed sheets. Advertising is a form of appeal writing, and this desire to read about things, when coupled with quotations, responds to some ineradicable desire of human nature. Nevertheless, as we all know, neither length nor quotation-making is sufficient to ensure a good appeal. Examples of the art are the pamphlets issued year by year from the Royal Hospital for Incurables, Putney Heath. We have had to praise them so often that really we are disappointed to find that the latest appeal offers only minor points for criticism. So long as this type of document is popular, so long as we feel its persuasive power, the institution deserves praise for these productions. "Suffering and Peace," as the latest is called, is a worthy successor to the series. With some of the quotations, that by Maeterlinck on the cover, for instance, we would quarrel at once; but then a quotation is either a challenge or a platitude, and, as we certainly prefer the former, it would be foolish to quarrel over this. We have recently criticised the system of election to this and other institutions. After reading these appeals we have no doubt that the skill which the management can command in their production should be sufficient to devise a more excellent way. The compilers of such appeals must be fully aware of the evils of petty patronage, and the attendant hunt for or purchase of votes. Why, then, are they doomed by the managers to carry them on? The present appeal is worthy of juster and saner system.

### THE EVOLUTION OF THE BATH-CHAIR.

Although the bath-chair has passed through various phases in design as modern ideas and requirements have dictated, its evolution can never be said to have been either startling or rapid; it is somewhat of a surprise therefore to hear of a motor bath-chair, but this is none the less true, since a Hampstead resident was recently fined at the local police court for driving such a vehicle without being in possession of the necessary licence. The chair appears to be of the "coach-built" type, suitably adapted with pneumatic tyres, etc., is electrically driven, and said to be capable of a speed of four miles per hour.

### SILHOUETTES IN CROSS-STITCH.

The most original feature in the issue of the *Gazette* of the 1st Eastern General Hospital, dated October 24, is a competition for sisters, nurses, and V.A.D.s. These are invited to make a souvenir of their stay in the hospital by working a series of silhouettes in cross-stitch round the border of an afternoon teacloth. The silhouettes, which represent hospital celebrities, will be judged at the next arts and crafts exhibition held at the hospital, when a prize will be given for the best sets. By way of illustration a silhouette of the O.C. is published, in the form of crosses to represent the stitches, in the present number, and these illustrations will continue until every hospital celebrity has been represented. It is suggested that the working of these silhouettes will provide a pleasant pastime for winter evenings, and we hope that many will find it so. The closing in of the open wards, to which allusion has already been made in The Hospital, is stated to be proceeding satisfactorily. We learn that "by making a wide central corridor the wards look considerably more spacious, and the stoves are not such an eyesore as had been feared."

### THIS WEEK'S DRUG MARKET.

With the exception of large transactions in quinine the drug market has been rather quiet; for some little time past the price of Continental brands of quinine had been steadily sagging until it reached a point at which buyers were tempted; the result was an unexpected rush of business at steadily advancing prices. The price of camphor has again advanced, and in view of the steady demand it is possible that prices have not yet reached the highest level. Opium is again rather dearer, and although prices of morphine are unchanged, makers are too busy to supply any large quantity promptly; holders of stocks are therefore able in some cases to sell morphine at prices rather above makers' quotations. Menthol is dearer. Castor oil and Epsom salts tend upwards in price. Citric acid and tartaric acid still have a downward tendency in value.

### TO OUR READERS.

**Contributions are specially invited from any of our readers to these columns. They should deal with topical subjects and news. They must be authenticated for the information of the Editor only. The minimum payment if published is 5s. There is no hard-and-fast rule as to space, but notes of about twenty lines in length are preferred.**

